# Microbiological Quality and Risk Assessment for Aflatoxins in Groundnuts and Roasted Cashew Nuts Meant for Human Consumption

**DOI:** 10.1155/2018/1308748

**Published:** 2018-06-03

**Authors:** Modupeade Christianah Adetunji, Ogechi Precious Alika, Ngozi Precious Awa, Olusegun Oladimeji Atanda, Mulunda Mwanza

**Affiliations:** ^1^Department of Animal Health, School of Agriculture, Faculty of Agriculture, Science and Technology, North-West University, Mafikeng Campus, Private Bag X2046, Mmabatho 2735, South Africa; ^2^Department of Biological Sciences, McPherson University, Km 96, Lagos-Ibadan Expressway, Seriki Sotayo, PMB 2094, Abeokuta, Ogun State, Nigeria

## Abstract

Nuts are one of the commonly consumed snacks but poor handling and storage practices can make them prone to foodborne infections. The study aimed at assessing the microbiological quality and risk assessment for aflatoxins in groundnuts and cashew nuts consumed in selected locations in Nigeria. The moisture content, colony counts, incidence of pathogenic bacteria, aflatoxin contamination, and risk assessment for aflatoxins were evaluated using standard methods. The moisture content and total viable count ranged from 5.00–8.60% and 5.5–89 × 10^3^ cfug^−1^, respectively, while the fungal count was between 4–24 × 10^3^ and 1.0–4.5 × 10^2^ cfug^−1^, respectively. Eleven fungal species belonging to 5 genera were isolated from the nuts, with* Aspergillus flavus, Rhizopus oryzae*, and* Fusarium oxysporum* having the highest percentage occurrence of 50%. In addition, the aflatoxin concentration ranged 0.1–6.8 and 29–33.78 ng kg^−1^ for cashew nuts and groundnuts, respectively. The margin of exposure (MOE) to aflatoxin contamination was 6.10 for groundnuts and 1000 for cashew nuts and the nuts consumers were at a risk of exposure to foodborne diseases and aflatoxin contamination with mean exposure values of 27.96 and 0.17 ng kg^−1^bwday^−1^, respectively. The risk of primary liver cancer for groundnuts and cashew nuts consumers was also estimated to be 1.38 and 0.01 canceryear^−1^100,000^−1^person, respectively. This calls for mitigation measures from appropriate governmental organizations.

## 1. Introduction

Nuts are delicacies generally accepted by almost everyone due to their high nutritive value and taste [1]. Apart from direct consumption, nuts are also useful for various industrial purposes such as extraction of oils for home and industrial uses, cooking, soap production, and body cream [2]. Nuts are rich in proteins, fats, minerals, and low water content; hence, they are highly susceptible to microbial invasion especially fungal attack [1].

Nuts are susceptible to fungal attacks at different stages and time. They may invade the nuts while still on the trees and this usually occurs when the hard shells or pods of the nuts are split open and the seeds attacked by insects or pests which creates room for the fungal spores to access the developing seeds. Other sources of contamination include harvesting, sorting, and washing of the nuts before storage. If the nuts are not properly handled at this stage, it could lead to mould growth especially when seeds are not properly dried to the recommended moisture level before storage [2, 3]. Another major source of mould invasion could be during storage when nuts are stored under conditions suitable for mould growth such as high temperature and humidity [4].

Both bacteria and fungi are responsible for microbial biodeterioration of food crops leading to food and economic losses, thereby reducing the export value of the food crops [5]. The fungal species could penetrate the hard shell of the cashew nut to commence biodeterioration processes without necessarily showing any form of mouldiness.

Mycotoxins are secondary metabolites produced by certain filamentous fungi [6, 7]. Aflatoxins are considered one of the main types of mycotoxins produced by fungi of the genus* Aspergillus,* especially* Aspergillus flavus* and* Aspergillus parasiticus.* Aflatoxins consumed by humans may lead to different forms of diseases like cancer, cirrhosis and other liver diseases, spontaneous abortion, and immunosuppression interference with micronutrient metabolism and stunted growth [8]. They have also been associated with diseases such as aflatoxicosis in livestock, domestic animals, and humans throughout the world. The adoption of proper measures for collection, storage, transportation, and handling could help reduce the risk of exposing nuts to attacks of mycotoxigenic fungi [9, 10]

Many countries have set maximum tolerable levels (MTLs) for mycotoxins of human health concern in foods, yet these regulations seem not to confer the required safety on the populace if large amount of foods with high susceptibility to aflatoxins such as maize, groundnuts, and melons are consumed by the population [11]. AFB_1_ has been implicated as a potent liver carcinogen, causing hepatocellular carcinoma (HCC) in humans and a variety of animal species. It has also been classified as Group 1 human carcinogen by the International Agency for Research on Cancer (IARC) with consequent negative effect such as adverse immune system disruption and stunted growth in children [12, 13]

Aflatoxins in foods are converted to aflatoxin-8,9-epoxide metabolite in the liver which seems to be responsible for many of the toxic effects in the body [14]. Coexposure to aflatoxins and hepatitis B virus (HBV) is common in developing countries and greatly increases HCC risk. Individuals with both exposures often have multiplicatively greater risk of developing HCC than those exposed to aflatoxins alone [15–17].

Despite the availability of literatures on mycotoxin contamination of groundnuts from different parts of Nigeria [18–20] information on the aflatoxin contamination and risk assessment due to consumption of cashew nuts is scarce. However, Adebajo and Diyaolu [21] reported on the incidence of aflatoxin and mould contamination of cashew nuts from Lagos State, Nigeria, but did not report on the risk of exposures of the consumers. This work is therefore aimed at assessing the microbiological quality and risk assessment for aflatoxins in cashew nut and groundnut consumers in Lagos and Ogun state, Nigeria. This will help to evaluate the current safety level of consumers in the selected states and also provide information to policy makers on appropriate line of actions to embark on.

## 2. Sample Collection and Preparation

Nuts are among the most common snacks consumed by almost everyone in Lagos State due to the busy nature of its inhabitants. Lagos is a cross metropolitan state with diverse kinds of migrants from within and outside the country who migrated into the state to earn a living. As a result of the busy schedule of the citizens, most people spend more time at work than at home and as a result eat less of homemade foods and more of snacks such as biscuits, nuts, and fried foods. Since nuts are perceived to be healthier and also have pleasant tastes, people have a great tendency to consume them instead of other snacks such as biscuits, chips, and popcorn. Due to the high population density of Lagos, new migrants now settle in Ogun state, a neighboring state to Lagos State, hence the choice of part of the state in the sampling process.

Twenty-seven samples of roasted cashew nuts and 15 samples of raw groundnuts were purchased from 9 and 5 locations in Lagos State and Obafemi Owode Local Government area, Ogun state, Nigeria. Samples were collected at random from three different traders in each location. Thus triplicate samples of roasted cashew nuts were purchased from the 5 zones that make up Lagos State, namely, “Eti-Osa” (“Ikota,” “Obalende,” and “Ajah”), “Badagry” (Market and “Topo”), “Ibeju-Lekki” (“Awoyaya and Lakowe”), “Mushin,” and “Ikeja,” while groundnut samples were purchased from the five major markets (“Ajebo,” “Fidiwo,” “Obafe,” “Ogunmakin,” and “Mowe”) in “Obafemi Owode” Local Government, Ogun State. Samples collected from the three different traders in each location were mixed together to form a composite sample/location. The composited samples were blended aseptically using Marlex Electronic Mixer Grinder (China) and 50 g portions kept in Ziplock envelopes. Samples that were not used immediately were kept at −20°C prior to analysis.

### 2.1. Moisture Content

Moisture content was determined out according to AOAC (2000) method. Briefly, samples (5 g) were oven-dried in a preweighed dish at 105°C for 4 h and cooled in a desiccator continuously until a constant weight was obtained. The moisture content was calculated from reduction in weight and expressed as a percentage of the original weight as illustrated below:

(1) $$\begin{array}{c} \%\text{ Moisture Content}=\frac{W_{2}-W_{3}}{W_{2}-W_{1}}\times 100, \end{array}$$

where

 
*W*_1_ is weight of empty Petri dish;
 
*W*_2_ is weight of sample + Petri dish before drying;
 
*W*_3_ is weight of sample + Petri dish after drying.

### 2.2. Determination of Total Viable and Fungal Counts

The total viable count (TVC) and fungal counts were determined by the dilution plate technique [22]. One gram of each blended nut was added to 9 ml of sterile water in a test tube and the solution was decimally diluted. Twenty millilitres of sterilized molten nutrient agar and Potato Dextrose Agar (PDA) plates supplemented with 0.01% chloramphenicol were cooled to 45°C and poured separately unto plates containing 1 ml aliquots of each sample in triplicate and the plates gently swirled and allowed to solidify. The nutrient agar plates were incubated at 30°C for 48 h for determination of TVC and Potato Dextrose agar plates at 28°C for 48 h for determination of fungal counts.

The total viable and fungal counts were determined using the following formula:

(2) cfu/g=Number  of  colonies×reciprocal  of  the  diluting  factorPlating  volume1 ml.

### 2.3. Isolation of Fungi by Direct Plating Method

This was determined using the method described by Adebajo and Diyaolu [21]. Seven whole nuts were obtained randomly for the assay of each nut. The cotyledons (2 per nuts) were hand separated, and the surfaces were disinfected with an aqueous solution of sodium hypochlorite 2% for 60 s. This was followed by three rinses in sterile distilled water, before four cotyledons were plated equispaced on the medium (PDA agar plus chloramphenicol 0.01%). The* Aspergillus* and* Penicillium* cultures on PDA were incubated unilluminated at 30 and 25°C, respectively, for 7 days. The observed colonies were purified on PDA agar plates to obtain pure cultures.

Identification of the fungal isolates was based on the examination of both the macroscopic characteristics (colony colour, morphology, and size) and microscopic characteristics (conidia morphology and size) features of the isolates on specific media and comparison with appropriate identification keys [18, 23, 24].

Isolates of* Aspergilli* (mainly black* Aspergillus*) on PDA were identified as* A. niger clade* [25]. The* Fusarium* and* Penicillium* isolates on PDA plates were also identified to genus level using appropriate manuals and references [22, 26, 27].

### 2.4. Determination of Aflatoxigenic Potential of Cashew Nut Isolates

Isolates belonging to the* Aspergilli* section Flavi were examined for aflatoxigenicity on Neutral Red Desiccated Coconut Agar (NRDCA), after 3 days of incubation by checking for fluorescence of agar under long wavelength UV light (365 nm).

The data obtained from toxigenic screening of* A. flavus* isolates on NRDCA was used to determine the ratio of aflatoxigenic isolates to nonaflatoxigenic isolates in the nut samples.

### 2.5. Determination of Percentage Fungal Occurrence

This was determined as described by Atanda et al. [28]. The percentage of fungal occurrence was calculated by dividing the occurrence of individual isolate per sample with the total number of all fungal isolates in the sample which was then expressed as a percentage using the following formula:

(3) $$\begin{array}{c} \frac{X}{Y}\times 100, \end{array}$$

where

 
*X* is total number of individual isolate per sample;
 
*Y* is total number of all fungal isolates per sample.

### 2.6. Test for Pathogenic Bacteria in Cashew Nuts

Aliquots (0.1 ml) of decimally diluted samples were spread plated on Mannitol salt agar at 32°C for 72 h for presumptive staphylococcal count. Colonies surrounded by bright yellow zones were counted. Confirmation of* Staphylococcus aureus* was a positive coagulase test [29].

The presumptive coliforms were counted on MacConkey agar No. 3 at 37°C for 24 h [29]. Confirmation of* Escherichia coli* was by fermentation of lactose and indole at 44°C after 24 h incubation.

### 2.7. Aflatoxin Determination by ELISA Method

Suspected aflatoxigenic fungi from both nuts were selected for aflatoxin analysis using the Enzyme Linked Immounosorbent Assay (ELISA) technique. Commercially available kit Agraquant®, total aflatoxin test kit (4–40 ng/kg), was used for the quantitative analysis of aflatoxins.

#### 2.7.1. Sample Preparation/Extraction

Five grams of the ground sample was weighed into a tightly sealed jar to which 25 ml methanol (70%) was added and allowed to settle for 5–10 min. The sample was then filtered using a No. 1 Whatman filter paper. Each of the fifty microlitres of the filtrate and aflatoxin standards in separate dilution wells was mixed with 100 *μ*l of the conjugate. Hundred microlitres was taken from the filtrate/standard-conjugate mixture and put in the antibody coated wells and incubated at room temperature (30°C) for 15 min. The contents of the wells were discarded and the wells washed with distilled water 3-4 times. Hundred microlitres of the substrate was added to each well and incubated for 5 min to allow for colour change (different shades of blue to colourless) and hundred microlitres of the stop solution was further added to each well to stop the reaction and this converted the blue end point to yellow and the wells were transferred to the ELISA reader machine to quantify the amount of toxins in the samples

#### 2.7.2. Quantification of Aflatoxins

This result was read at 450 nm, using ELISA plate reader. Optical densities of standards (0 ngg^−1^, 4 ngg^−1^, 10 ngg^−1^, 20 ngg^−1^, and 40 ngg^−1^) and those of samples were also recorded. A graph of the standard concentration versus optical densities was plotted and extrapolations were made to determine levels of total aflatoxins in the roasted cashew nuts samples.

### 2.8. Exposure Assessment of Nut Consumers to Aflatoxin Contamination

The exposure levels of the nut consumers were determined as described by by Adetunji et al. [30]. The quantity of aflatoxins in the groundnut samples was multiplied by the average consumption rate of the nuts in Nigeria (groundnuts, 52 g/person/day) according to WHO [31] which was then divided by the average body weight of 60 kg for adults [30]. There is paucity of information in literature on the average daily consumption rate of cashew nuts in Nigeria; hence the average consumption rate for cashew nuts was extrapolated by comparing the ratio of annual production of the two nuts. The annual production of groundnuts was reported as 3,028,571 tons (http://www.factfish.com/statisticcountry/nigeria/peanuts%2C%20production%20quantity) while that of cashew nuts was 120,000 tons (http://agriculturenigeria.com/farming-production/crop-production/cash-crops/cashew), thus with a production differential ratio of approximately 1 : 25. Thus, the consumption rate for cashew nuts in this report was estimated as 2.08 g person^−1^day^−1^, obtained by dividing the average daily adult consumption rate for groundnuts with the production differential ratio of the two nuts. The exposure assessment (PDI) was thus calculated as illustrated below:

(4) $$\begin{array}{c} PDI_{m}=\frac{C_{m}\times C_{c}}{Bw}, \end{array}$$

where

 
PDI_*m*_ is probable daily intake for aflatoxins (ngkg^−1^bwday^−1^);
 
*C*_*m*_ is mean level of aflatoxins in the nuts per location (ng g^−1^);
 
*C*_*c*_ is average consumption of nuts in Nigeria (g day^−1^);
 
Bw is body weight for an adult (kg).

### 2.9. Risk Characterization for Aflatoxins

Risk characterization for genotoxic and carcinogenic compounds such as aflatoxins is based on the margin of exposures (MOEs), which was calculated by dividing the Benchmark dose lower limit (BMDL) for aflatoxins- 170 ngkg^−1^bwday^−1^ [32] by the toxin exposure.

In cases where MOEs were lower than 10,000, a public health concern is indicated which implied that aflatoxin exposures above 0.017 ngkg^−1^bwday^−1^ (as obtained by dividing 170 ngkg^−1^bwday^−1^ by 10,000) represented a risk of public health concern [33].

### 2.10. Estimated Liver Cancer Risk due to Consumption of Nuts

The estimated liver cancer risk for Nigerian adult consumers was calculated for aflatoxins because the ingestion of the toxin can be traced to the development of liver cancer [32, 34]. This involved estimating the population cancer risk per 100,000 which was obtained by multiplying the PDI_*m*_ value with the average hepatocellular carcinoma (HCC) potency figure from individual potencies of HBsAg-positive and for HBsAg-negative groups.

The JECFA estimated potency values for AFB_1_ which corresponded to 0.3 cancersyear^−1^100,000^−1^population/ngkg^−1^bwday^−1^ (uncertainty range: 0.05–0.5) in HBsAg-positive individuals and 0.01 cancersyear^−1^100,000^−1^population/ngkg^−1^bwday^−1^ (uncertainty range: 0.002–0.03) in HBsAg-negative individuals [32, 34] were adopted for this calculation. Also, the HBsAg+ prevalence rate of 13.6% for Nigeria [35] was adopted and 86.4%  (100 − 13.6%) was extrapolated for HBsAg-negative groups. Hence the average potency for cancer in Nigeria was estimated as follows:

(5) Average  potency=0.3×0.136+0.01×0.864=0.04944 cancers per year per 100,000 population per ng  AFB1  kg−1bwday−1.

Thus the population risk was calculated using the following formula:

(6) $$\begin{array}{c} \text{Population risk}=\text{Exposure}\times \text{Average potency}. \end{array}$$

### 2.11. Statistical Analyses

The data obtained from the analysis were reported as means of three replicates and subjected to analysis of variance (ANOVA) at 95% confidence level to test for significance using SPSS 16.0 software (Windows version, SPSS, IL, USA). Colony counts of nuts were calculated as cfu/g. The relationship between aflatoxigenicity of* Aspergillus* isolates and aflatoxin concentrations in cashew nuts was evaluated using Pearson correlation analysis.

## 3. Results

### 3.1. Microbiological Quality of Cashew Nut

#### 3.1.1. Moisture Content and Total Viable Count

The average moisture content of the roasted cashew nuts from Lagos State ranged 5.0–8.6% and were not significantly different (*P* ≥ 0.05) from each other. The mean moisture content of cashew nuts was 6.04% with “Ajah” (5.0%) and “Obalende” (8.6%), having the lowest and highest moisture content. The groundnut samples from Ogun state had an average moisture content of 6.12%.

Roasted cashew nuts from “Ikeja” had the highest TVC count (64.5 × 10^3^ cfug^−1^) while “Obalende” had the lowest count of 5.5 × 10^3^. Furthermore, a mean TVC count of 29.67 × 10^3^ cfug^−1^ was recorded for cashew nuts from Lagos State while the mean viable bacterial count for the groundnuts was 71.20 × 10^3^ cfug^−1^ with “Mowe,” having the highest viable count of 89.00 × 10^3^ cfug^−1^. The fungal counts also ranged 1.0 × 10^2^ cfug^−1^–4.5 × 10^2^ cfug^−1^ with a mean count of 2.30 × 10^2^ cfug^−1^ for cashew nuts while groundnuts had counts that ranged from 4.00 × 10^3^ cfug^−1^ for “Fidiwo” to 24.00 × 10^3^ cfug^−1^ for “Mowe” (Table 1).

#### 3.1.2. Incidence of Pathogenic Bacteria in Roasted Cashew Nuts

The incidence of pathogenic bacteria in the roasted cashew nuts is shown in Table 2. Coliforms were present in most samples in Lagos State except for those collected from “Ajah,” while* Staphylococcus aureus* was found in cashew nuts from only 2 locations (“Awoyaya” and “Badagry”) out of the 9 sampled locations (22.22%) in the state. “Topo” had the highest* coliform* count of 12 × 10^2^ cfug^−1^ while “Obalende” and “Badagry” had the lowest counts of 1 × 10^2^ cfug^−1^.

#### 3.1.3. Fungal Occurrence

Nine fungal species belonging to 5 major genera were isolated from the roasted cashew nuts in Lagos State while 7 were isolated from the groundnut samples (Table 3). The common organisms to both nuts were as follows:* Aspergillus carbonarius*,* Aspergillus niger*,* Aspergillus flavus*,* Penicillium *sp., and* Curvularia lunata. Fusarium *spp. was not found in the groundnut samples. The dominant fungi found in Lagos State were as follows:* Aspergillus flavus, Rhizopus oryzae*, and* Fusarium oxysporum* with 50% occurrence rates. “Lakowe” had the highest occurrence of fungal species (55.55%) while samples from “Badagry” and “Ajah” had the least occurrence (9%). The dominant fungi in the groundnut samples were* A. flavus* and* A. niger* with 60% occurrence each.

### 3.2. Aflatoxin Producing Potential of Cashew Nut Isolates

The aflatoxigenicity test carried out on the* Aspergillus* isolates from the cashew nuts showed that only* A. flavus species *(55.55%) had the potential to produce aflatoxins (Table 4). The positive isolates were stored in autoclaved glycerol water (10% glycerol) in an Eppendorf tube and stored at −80°C for subsequent use.

### 3.3. Aflatoxins Concentration of Nuts

The aflatoxin concentration of the cashew nuts ranged from 0.1 ngg^−1^ to 6.8 ngg^−1^ with “Ikeja” and “Lakowe” having the least (0.1 ngg^−1^) and highest (6.8 ngg^−1^) concentrations while the aflatoxin concentration of the groundnuts ranged 29–33.78 ngg^−1^ (Table 5) with “Ajebo” having the least (29 ngg^−1^) and “Mowe” (33.78 ngg^−1^) the highest aflatoxins concentration.

### 3.4. Correlation between Aflatoxigenic Potential of Fungal Isolates and Aflatoxin Concentration of Cashew Nuts

A significant (*P* < 0.01) inverse correlation (*r* = −0.980) existed between aflatoxin producing potential of* Aspergillus flavus* on NRDCA and the aflatoxin concentration of cashew nuts from the different locations (Table 6).

### 3.5. Exposure Assessment of Nuts Consumers to Aflatoxins

The range of exposures of consumers to aflatoxin contamination due to consumption of groundnuts in Obafemi Owode Local Government, Ogun State, and cashew nuts in Lagos State ranged 25.13–29.28 ngkg^−1^bwday^−1^ (Table 7) and 0.00–0.24 ngkg^−1^bwday^−1^(Table 8) with a mean of 27.96 and 0.17 ngkg^−1^bwday^−1^, respectively, which were higher than the permissible exposure level of 0.017 ngkg^−1^bwday^−1^. The margin of exposures of the groundnut consumers (6.10) as compared with MOE of cashew nut consumers (1000) was much lower than the permissible limit of 10,000 (Tables 7 and 8).

Furthermore, the HCC prevalence rate for groundnut consumers in Obafemi Owode Local Government, Ogun State, ranged 1.24–1.45 cases 100,000^−1^peopleyear^−1^ with a mean value of 1.38 cases 100,000^−1^peopleyear^−1^ while there is little or no risk of HCC (0.00–0.01 cases 100,000^−1^peopleyear^−1^) for the cashew nut consumers in Lagos State.

## 4. Discussion

The moisture contents of the roasted cashew nuts were above the permissible recommended moisture limit of 5% [36]. Similarly the higher moisture content of the groundnut samples (6.12%) could be as a result of its raw nature as it has not yet undergone any form of processing. A similar range of moisture content (6.48–7.05%) was reported for groundnuts from different agroecological zones of Nigeria by Oyedele et al. [18]. However, the moisture content of our roasted cashew nuts was higher than the findings of Adebajo and Diyaolu [21] who reported a range of moisture content of 4.1–6.8% with a mean of 5.4% in cashew nuts from the same state. Oluwafemi et al. [5] also reported a lower moisture content range of 3.7–4.3 and 3.2–5.4% for cashew nuts from Ogun state during the dry and raining seasons.

The high moisture contents of the samples may also be as a result of improper packaging or lack of use of good packaging materials. Nuts are colloids materials (hygroscopic), which tend to absorb moisture from the surrounding atmosphere until it reaches equilibrium, Oladapo et al. [37]. The moisture levels of the nuts could also be influenced by the harvesting methods of the farmers.

The difference in the microbial load of some of the samples may be due to the varying climatic conditions in the different zones across the two states. “Ikota,” “Ajah,” and “Obalende” (“Eti-Osa” zone) are situated in the humid forest zones of Lagos State. The high humidity in this zone may be a contributing factor to the higher microbial counts of this zone and probably coupled with improper drying, poor handling, poor processing techniques, and storage methods of the nuts. Several authors had reported a dense population of fungi in groundnut samples [2, 18, 38] which also corroborated the findings of this research work. The bacteria and fungi load of the cashew nuts were however less than the 10^3^ cfu/g and 10^5^ cfu/g limits recommended in foods by the International Commission on Microbiological Specification for Foods (ICMSF, 1998). The lower counts of viable organisms in the cashew nuts may be due to the presence of anacardic acids and phenolic compounds present in cashew nut oils which are inhibitory action against Gram-positive bacteria, yeast, and fungi. Anacardic acids with distinct lateral chains have been shown to be effective against the growth of* Staphylococcus aureus, Propionibacterium *spp.*, Streptococcus mutans*, and* Brevibacterium ammoniagenes *[39]. Although the microbial counts of the nuts were lower than the recommended limit at the time of purchase and analysis, there is the possibility of probable increase in the microbial load of the remaining market samples due to poor handling practices of the products by the sellers. A larger percentage of the samples were purchased from hawkers who vended the products in polyethylene plastics in the sun. Continual exposure of the nuts to the sun during the day and recondensation in the evenings could support increase in the microbial load of the samples. With improper management of storage temperatures and humidity, aflatoxins producing moulds may rapidly reproduce representing a food safety problem and a major risk for the production of aflatoxins. Furthermore,* Aspergillus *spp. can easily grow at optimal conditions of 27–33°C, pH range of 5-6 and water activity of 0.82–0.99 [40]. Contamination of nuts by moulds may also occur early in the field and deterioration could develop during prolonged storage leading to the production of mycotoxins [41].

The high incidence of* A. flavus, A. niger, Penicillium *spp*., *and* Rhizopus oryzae* and scant occurrence of* Fusarium *spp. reported in our nuts complemented the report of previous authors [2, 38], who reported a similar trend of fungal occurrence; however, Oyedele et al. [18] reported a higher incidence of* Fusarium *spp. (45%) in groundnut samples across the agroecological zones of Nigeria.

Although cashew nut is known to be an excellent substrate for microbial growth, outbreak of serious infections through its consumption had rarely been recorded. However, the probable presence of* E. coli* in the samples from Lagos State is a source of concern as it is transmitted to humans primarily through consumption of contaminated foods. Symptoms of the diseases caused by this Shiga toxin-producing* E. coli* include abdominal cramps and diarrhoea that may in some cases progress to bloody diarrhoea (haemorrhagic colitis). Fever and vomiting may also occur.

Staphylococcal food poisoning is also one of the most common causes of reported foodborne diseases. Contaminated equipment and environmental surfaces also can lead to* S. aureus* infections whose symptoms include diarrhoea, vomiting, nausea, and abdominal cramping. Freire and Offord [41] and Oluwafemi et al. [5] had earlier reported on higher counts of* S. aureus* in Brazilian (5.3 × 10^3^–1.2 × 10^4^ cfug^−1^) and Nigerian (1–14 × 10^4^ cfug^−1^) roasted cashew nuts, respectively. Processing conditions such as roasting and salting could be responsible for the low count of* S. aureus* in our samples in addition to the presence of anacardic acid.* Staphylococcus aureus* is the most osmotolerant foodborne pathogen and the outbreaks of staphylococcal food poisoning are often linked to foods of reduced water activities [5, 42] and this could lead to septicaemia in humans. It is ideal that nut vendors inculcate simple sanitary habits such as proper disposal of fecal waste and constant washing of hands with clean water and soap especially after visiting the toilet or in contact with any fecal objects. Food processors and handlers are also advised to stay away from processing foods when they have cold, flu, cough, and diarrhoea to avoid contamination of foods with the causative organisms.

This report further reemphasizes the suitability of NRDCA for screening of toxigenic fung in foods before further analyses with HPLC or other sophisticated methods. It is also readily more available as it can be easily composited as described by Atanda et al. [28]

Eighty percent (80%) of the tested samples had aflatoxin concentrations above the European Union (EU) permissible limit of 4 *μ*g/kg, while 100% of the groundnut samples had aflatoxin concentration higher than the recommended 20 *μ*gkg^−1^ permissible limit set by the Standard Organization of Nigeria for unprocessed raw groundnuts. Similarly, higher concentration of aflatoxins (11.33–20.67 *μ*gkg^−1^) was reported for cashew nuts from Venezuela [43] while a lower concentration of AFB_1_ (0.26–0.32 *μ*gkg^−1^) and total aflatoxins (0.5–0.84 *μ*gkg^−1^) was reported for imported cashew nuts from Istanbul, Turkey [44]. A higher aflatoxin concentration was however reported for groundnut samples from Kenya (Max; 250 *μ*gkg^−1^), Iraq (10.3 *μ*gkg^−1^), and Nigeria (216.1 *μ*gkg^−1^) [2, 18, 38]. Aflatoxins are highly toxic, fungal metabolites commonly known to adversely affect the health and nutrition status of humans and other animals in various ways. High concentration of aflatoxins is most often found in plants with very nutritive seeds such as maize, nuts, and cereal grains in Africa [45]. Symptoms of aflatoxicosis are gastrointestinal infections including vomiting and abdominal pain. The toxin had also been incriminated as a potential mutagenic, carcinogenic, and teratogenic threat to humans [46].

The high concentration of aflatoxins in the nuts especially groundnuts is reflected in the high PDI and MOE values of the nuts. Groundnut consumers in the local government state are therefore at high risk of exposures to aflatoxicosis and liver cancer and other forms of disabilities due to consumption of the contaminated nuts. The evaluation of risk assessments of aflatoxins by international bodies has been based on the carcinogenic potencies developed by JECFA, but, recently, the EC Scientific Panel on Contaminants in the Food Chain [32] developed a benchmark dose (BMD) approach. The BMD, which is a mathematical model originally put forward by Crump [47], represents an estimate of the dose required to produce a small response (1–10%) above the control. The BMD lower limit of 170 ngkg^−1^bwday^−1^ refers to the corresponding lower limits of a one-sided 95% confidence interval on the BMD. For the evaluation of human and experimental animal data, the EFSA Scientific Committee thus proposed the use of the BMD methodology to derive a reference point on the dose-response curve. In addition, the BMD approach is recommended in the US EPA's Proposed Guidelines for Carcinogen Risk Assessment [48] regarding modelling tumour data and other (noncancer) responses thought to be important precursor events in the carcinogenic process.

Although the risk of exposure through cashew nut consumption was low in Lagos State, there is still cause for concern because the smallest molecule of the aflatoxin metabolite could evoke changes in human DNA leading to mutations, selective cellular proliferation, and cancer [49]. Aflatoxin B_1_ has a reactive metabolite that interacts directly with DNA; hence, it is assumed that there is no safe dose above zero. The recommendation of JECFA with regard to safe level of aflatoxins in foods is to reduce “As Low As Reasonably Achievable (ALARA)” considering the remarkable genotoxic carcinogenic potential of this toxin [50]. The report of our findings corroborates the report of Oyedele et. al., 2017, who also reported a high PDI value of 91.2 ngkg^−1^bwday^−1^ for groundnut consumers in the humid forest zones of Nigeria. A recent survey of the occurrence of AFB_1_ in Malaysian groundnuts also revealed that Malaysians are exposed to high concentrations (24.3–34.0 ngkg^−1^bwday^−1^) of AFB_1_ due to high daily consumption rate of groundnuts in their diets [51]. Kooprasertying et al., 2016 [52], also reported high aflatoxin concentrations of peanuts sold in Thailand but at lower exposure risks (0.36–1.91 ngg^−1^). However, the rate of exposure to aflatoxins through consumption of aflatoxin contaminated nuts in developed countries is minimal when compared to the situation in developing and underdeveloped countries like Nigeria.

Liver cancer is ranked as the second and third common cancer in men and women, respectively, and the leading cause of deaths in men and the third leading cause of cancer deaths in Africa women [53]. In 2012, 12,047 cases of liver cancer were estimated for Nigeria with age standardized incidence rates (ASR) of 11.5 per 100,000 for all causes of cancer [54]. Thus it can be estimated from our result that eating aflatoxin contaminated foods could be responsible for 12% of all cancer cases in Nigeria (1.38/11.5 × 100).

It was reported that the maximum tolerable limit for aflatoxins permitted in foods for liver cancer risk to be increased by 1 out of 100,000 and 10,000 population in Nigeria in a lifetime is 2 ppb and 24 ppb, respectively [11]. The concentrations of aflatoxins in our groundnuts were higher than the 2 ppb; thus a large percentage of the consumers are at a risk of hepatocellular carcinoma. In addition, the MOE values which were very low for the groundnut consumers in Ogun state indicated a public health threat to the populace. Our findings also showed that consumers of cashew nuts in Lagos State are at low risk of primary liver cancer

Several authors had reported the existence of a positive correlation between aflatoxin exposure and HCC in HBV positive individuals [16, 35, 55]. The global burden of HCC induced by aflatoxins was estimated to be 4.6–28.2% [16] and most cases occur in sub-Saharan Africa, Southeast Asia, and China, where populations suffer from both high HBV prevalence and largely uncontrolled exposure to aflatoxins in foods. An assessment carried out by the Joint FAO/WHO Expert Committee on Food Additives (JECFA) to estimate the impact of aflatoxins exposure on HCC incidence revealed that if the hypothetical total aflatoxins permissible standard of 20 *μ*gkg^−1^ was reduced to a stricter standard of 10 *μ*gkg^−1^, HCC incidence would decrease by about 300 cases per billion people per year in countries with an HBV prevalence of 25% [56, 57].

The inverse correlation between toxigenic isolates and aflatoxin concentrations confirms the fact that certain requirements (suitable temperature, water activity, relative humidity, and storage environment) must be met before the elaboration of aflatoxins in the food substances [40].

## 5. Conclusion

This study showed that the nuts were contaminated with pathogenic bacteria and toxigenic fungi, resulting in aflatoxin contamination of the products, thereby posing health risks to their consumers. There is need for improvement of sanitary practices, regular monitoring, and enforcement of standards for foods sold in open markets in order to reduce the menace of food borne diseases and mycotoxicoses among the populace. In addition, proper packaging materials and good storage practices should be enforced.

## Figures and Tables

**Table 1:** Moisture content and colony counts of groundnuts and cashew nuts.

Groundnut				Cashew nut			
Location	Moisture content	Total viable Count (×10^3^ Cfu/g)	Fungal count (×10^3^ Cfu/g)	Location	Moisture Content (%)	Total viable count (×10^3 ^cfu/g)	Fungal count (×10^2^ cfu/g)
“Ajebo”	5.10^a^	70.00^a^	5.00^a^	“Mushin”	5.2^a^	37.5^d^	3.0^a^
“Fidiwo”	6.12^a^	58.00^a^	4.00^a^	“Ajah”	5.0^a^	25.0^c^	4.5^b^
“Obafe”	5.63^a^	78.00^b^	24.00^c^	“Ikota”	5.3^a^	9.5^b^	1.0^a^
“Ogunmakin”	6.57^a^	61.00^a^	4.00^a^	“Lakowe”	6.1^a^	13.5^b^	1.5^a^
“Mowe”	7.2^a^	89.00^b^	10.00^b^	“Awoyaya”	6.3^a^	49.5^e^	4.0^b^
				“Badagry”	5.6^a^	52.5^e^	1.5^a^
				“Topo”	5.8^a^	9.5^b^	1.2^a^
				“Obalende”	8.6^b^	5.5^a^	1.5^a^
				“Ikeja”	6.5^a^	64.5^f^	2.5^a^
Mean	6.12	71.2	9.40	Mean	6.04	29.67	2.30

Mean values with the same superscript along the same column are not significantly different (*P* < 0.05).

**Table 2:** Incidence of pathogenic bacteria in roasted cashew nuts from Lagos State, Nigeria.

Market	*Staphyloncus aureus *count (×10^2^ cfu/g)	Coliform count (×10^2^ cfu/g)
“Lakowe”	0	4^a^
“Topo”	0	12^b^
“Obalende”	0	1^a^
“Ikota”	0	10^b^
“Awoyaya”	2^a^	3^a^
“Badagry”	1^a^	1^a^
“Ajah”	0	-
“Mushin”	0	2^a^
“Ikeja”	0	2^a^

Mean values with the different superscripts along the same column are significantly different (*P* ≥ 0.05).

**Table 3:** Percentage occurrence of fungal isolates in Nigerian nuts.

Sample type	Location	Fungal Isolate (%)										
		*A. flavus*	*A. niger*	*A. Parasiticus*	*A*. *fumigatus*	*Penicillium*spp.	*A. candidus*	*A. carbonarius*	*Curvularia lunata*	*Rhizopus oryzae*	*Fusarium oxysporum*	*Fusarium verticillioides*
Cashew nut	“Mushin”	ND	33.3^a^	ND	ND	ND	ND	ND	ND	ND	^*∗*^66.7^b^	ND
	“Topo”	ND	ND	ND	ND	ND	ND	25^a^	ND	50^b^	ND	25^a^
	“Awoyaya”	25^a^	ND	ND	25^a^	25^a^	ND	ND	ND	ND	ND	25^a^
	“Lakowe”	16.7^a^	ND	ND	33.3^b^	ND	ND	16.7^a^	ND	16.7^a^	16.7^a^	ND
	“Obalende”	25^a^	25^a^	ND	ND	ND	ND	ND	25^a^	25^a^	ND	ND
	“Badagry”	ND	100	ND	ND	ND	ND	ND	ND	ND	ND	ND
	“Ikota”	25^a^	25^a^	ND	ND	ND	ND	ND	ND	25^a^	25^a^	ND
	“Ajah”	ND	ND	ND	ND	ND	ND	ND	ND	ND	100^a^	ND
^*∗∗*^Overall occurrence (%)		50	37.5	ND	12.5	12.5	ND	25	12.5	50	50	25
Groundnut	“Ajebo”	28.57	42.85	ND	ND	ND	ND	14.28	14.28	ND	ND	ND
	“Fidiwo”	83.33	ND	16.67	ND	ND	ND	ND	ND	ND	ND	ND
	“Obafe”	4.76	4.76	ND	ND	80.95	ND	ND	9.52	ND	ND	ND
	“Ogunmakin”	ND	100.00	ND	ND	ND	ND	ND	ND	ND	ND	ND
	“Mowe”	ND	ND	ND	ND	87.50	12.5	ND	ND	ND	ND	ND
Overall occurrence (%)		60	60	20	ND	40	20	20	40	ND	ND	ND

ND: not detected; mean occurrence with same superscript along the same row is not significantly different (*P* ≥ 0.05). ^*∗*^Frequency of occurrence = (total number of each organism in the sample )/(total number of all organisms in the sample). ^*∗∗*^Overall occurrence = (no. of locations that each organism is found /(total no. of locations sampled).

**Table 4:** Aflatoxigenicity of fungal isolates from cashew nuts from Lagos State on Neutral Red Desiccated Coconut Agar (NRDCA).

Location	Aflatoxin producing potential

“Ikeja”	Negative
“Obalende”	Positive
“Mushin”	Negative
“Badagry” Market	Positive
“Ikota”	Positive
“Mushin”	Positive
“Lakowe”	Positive

**Table 5:** Aflatoxin concentration of nuts.

Cashew nut		Groundnut	
Location	Aflatoxin concentration (ng/g)	Location	Aflatoxin concentration (ng/g)
“Lakowe”	6.80^b^	“Ajebo”	29.00^a^
“Obalende”	5.80^b^	“Fidiwo”	33.00^a^
“Ikota”	5.40^b^	“Obafe”	32.64^a^
“Ikeja”	0.1^a^	“Ogunmakin”	32.88^a^
“Badagry”	6.4^b^	“Mowe”	33.78^a^

Mean concentrations with same superscript along the same column are not significantly different (*p* ≥ 0.05).

**Table 6:** Correlations analysis between aflatoxins concentration and aflatoxigenic potential of isolate.

	Aflatoxin concentration	Aflatoxin potential
Aflatoxin concentration		
Pearson correlation	1	−.980^*∗∗*^
Sig. (1-tailed)		.002
*N*	5	5
Aflatoxin potential		
Pearson correlation	−.980^*∗∗*^	1
Sig. (1-tailed)	.002	
*N*	5	5

^*∗∗*^Correlation is significant at the 0.01 level (1-tailed). *r* = −.980, *N* = 5, *P* < 0.01.

**Table 7:** Risk Assessment due to consumption of groundnuts from Obafemi Owode Local Government Area, Ogun State, Nigeria.

Location	Mean aflatoxin concentration (ngg^−1^)	^1^PDI (ngkg^−1^bwday^−1^)	^2^Margin of exposure (MOE)	^3^Population risk for primary liver Cancer (canceryear^−1^100,000^−1^ population)
“Ajebo”	29.00^a^	25.13	6.76	1.24
“Fidiwo”	33.00^a^	28.60	5.94	1.41
“Obafe”	32.64^a^	28.29	6.01	1.40
“Ogunmakin”	32.88^a^	28.50	5.97	1.41
“Mowe”	33.78^a^	29.28	5.81	1.45
Mean^4^	32.26	27.96	6.10	1.38

Values with different superscripts along the same column are significantly different at *P* < 0.05. ^1^Probable Daily Intake (PDI) calculated by multiplying the aflatoxin concentration in groundnuts by consumption rates of groundnuts in Nigeria (52 g/person/day). ^2^Margin of exposure (MOE) calculated by dividing the benchmark dose lower limit (BMDL) for aflatoxins by AFB1 exposure. ^3^Population risk for primary liver cancer = exposure × average potency (0.04944). ^4^Mean of aflatoxin concentration.

**Table 8:** Risk Assessment due to consumption of cashew nuts from Lagos State, Nigeria.

Location	Mean aflatoxin (ngg^−1^)	^1^PDI (ngkg^−1^bwday^−1^)	^2^Margin of exposure (MOE)	^3^Population risk for primary liver cancer (canceryear^−1^100,000^−1^ population)
“Lakowe”	6.80^b^	0.24	708.33	0.01
“Obalende”	5.80^b^	0.20	850.00	0.01
“Ikota”	5.40^b^	0.19	894.73	0.01
“Ikeja”	0.1^a^	0.00	0.00	0.00
“Badagry”	6.4^b^	0.22	772.72	0.01
^4^Mean	4.9	0.17	1000	0.01

Values with different superscripts along the same column are significantly different at *P* < 0.05.  ^1^Probable Daily Intake (PDI) calculated by multiplying the aflatoxin concentration in cashew nuts by consumption rates of cashew nuts in Nigeria (2.08 g/person/day) as estimated by WHO 2008 and divided by assumed body weight for Nigerian adults (60 kg).  ^2^Margin of exposure (MOE) calculated by dividing the benchmark dose lower limit (BMDL) for aflatoxins by AFB_1_ exposure. ^3^Population risk for primary liver cancer = exposure × average potency (0.04944).  ^4^Mean of aflatoxin concentration.
